# Mortality of Surgical Operations in the Upper Lake States, Compared with That of Other Regions

**Published:** 1876-11

**Authors:** Edmund Andrews

**Affiliations:** Professor of Princples and Practice of Surgery in Chicago Medical College


### THE MORTALITY OF SURGICAL OPERATIONS IN THE UPPER LAKE STATES, COMPARED WITH THAT OF OTHER REGIONS.

By EDMUND ANDREWS, A.M., M.D.,

Professor of Principles and Practice of Surgery in Chicago Medical College,

Assisted by Thos. B. Lacey, M.D.,

Assistant Surgeon in the National Soldiers' Home.

(Continued.)

### OTHER AMPUTATIONS OF THE FOOT.

I have included these all in a single table, which is here subjoined:

### TABLE XI.

### AMPUTATIONS THROUGH TARSUS AND METATARSUS IN LAKE STATES, (EXCLUDING PIROGOFF'S AND SYME'S.)

| No. | OPERATOR OR REPORTER. | Age. | CAUSE OF OPERATION. | COMPLICATIONS. | OPERATION. | Con-diti'n | Time of operation | Result. | Time to death or recovery | Practice. |
|---|---|---|---|---|---|---|---|---|---|---|
| 6654 | Dr. E. Andrews | 24 | Crushed toes | ---- | In metatarsus | Good | 12 days | Recov'r'd | 6 weeks. | Hospital. |
| 5129 | " " | -- | Both feet frozen | ---- | At juncture of tarsus and metatarsus of both feet | " | Second'y. | " | 3 mos. | " |
| ---- | ---- | -- | Fracture of both feet | ---- | At juncture of tarsus and m tatarsus of both feet | ---- | " | " | ---- | " |
| ---- | " Hyde | 32 | Feet frozen | None | Junct. tarsus & metatarsus | Med. | " | " | 1 month | " |
| ---- | " E. Owens | -- | ---- | ---- | Chopart's in one foot and Hey's in the other | ---- | " | " | ---- | " |
| ---- | " H. Wardner | 24 | Frost bite | Drunk | Both feet through metatar. | Bad | Primary | " | 3 mos. | Private. |
| ---- | " " | 18 | Foot crushed | None | Chopart's | Good | " | " | 4 " | " |
| ---- | " " | 16 | " " on R. R | Consider'ble shock | " | " | " | " | 5 " | " |
| ---- | " " | 12 | " " " | ---- | " | Med. | " | " | 6 " | " |
| ---- | " " | 30 | Feet frost bitten and mortified | ---- | Metatarsus of one foot and all the toes of the other. | Bad | 8 days | Died | 3 days | " |
| ---- | " E. D. Kittoe | 22 | Foot cut off by axe | Great shock | Chopart's | Good | 3 days | Recov'r'd | ---- | " |
| ---- | " " | 23 | Foot crushed by stone | Up. & lo. jaws fract. | " | Med. | 6 hours | " | ---- | " |
| ---- | " J. Andrews. | 30 | Wound of dorsal artery of foot | Caries of foot | " | Bad | Second'y. | " | ---- | " |
| ---- | " E. W. Lee | 19 | Foot crushed by R. R | None | " | Good | 4 hours | " | 4 weeks. | " |
| ---- | " " | 18 | " " " | " | Metatarso-phalangeal articulation | " | 3 hours | " | 5 " | " |
| ---- | " S. Marks | 24 | " " | ---- | Chopart's | " | Primary | " | 2 mos. | Hospital. |
| ---- | " " | 31 | " " | ---- | " | " | " | " | 3 " | Private. |

RECAPITULATION.

| | CASES. | DIED. | PER CENT. MORTALITY. |
|---|---|---|---|
| Total of all kinds | 17 | 1 | 6 |
| Total Chopart's amputation | 10 | 0 | 0 |
| Chopart's primary | 7 | 0 | 0 |
| " intermediary and secondary | 3 | 0 | 0 |
| Junction of tarsus and metatarsus | 3 | 0 | 0 |

### CHOPART'S AMPUTATION ABROAD.

The few published statistics of this operation are imperfectly classified. Of 101 cases of all kinds, sixteen died, which is about sixteen per cent., while of our ten cases recorded in the Lake States none died.

### OPINIONS OF AUTHORS.

As above stated, Demme, Stromeyer and Legouest show military statistics to the effect that in compound gunshot fractures of the foot, conservative treatment is better by a large figure than any amputation.

Holmes' System of Surgery, Vol. V., p. 642, says Chopart's amputation is "undesirable," on account of the tendency of the stump to point its extremity too much downward.

Erichsen, Vol. I., p. 73, thinks that Hey's amputation at the tarso-metatarsal articulation is often not desirable, and prefers if possible to saw through the metatarsals in front of the joint. In respect to Chopart's amputation he, contrary to Holmes' opinion, says that the stump is excellent, and that if it points too much downward it should be remedied by division of the tendon of Achilles. He advises to saw off the head of the astragalus, and the articular surface of the os calcis.

### GENERAL EFFECTS OF AGE, SEX, TIME OF OPERATION, AND PATHOLOGICAL CONDITION ON THE MORTALITY OF AMPUTATIONS.

These topics are as yet very imperfectly investigated, owing to the present rude condition of the science of statistics. Some dim light, however, has been thrown on them.

AGE.—Malgaigne's statistics of the Hospitals of Paris give the following conclusions;

1. Under five years of age the mortality of amputations is more than between five and fifteen years.

2. From five to fifteen years is the most favorable age.

3. From fifteen to twenty years the risk increases.

4. From twenty to fifty years it remains nearly stationary, but increases again after fifty. (Dictionnaire des Sciences Médicales.)

Mr. Callender, of St. Bartholomew's Hospital, London, says that in that institution the death of a child or of a patient under the age of forty is an exception, and that age increases the tendency to death.

From 1853 to 1863 the mortality of primary amputations all ages was less than ten per cent., while of ten cases over sixty-five years of age, sixty per cent. died. The experience of St. Bartholomew's Hospital is very valuable, because it is of all the hospitals in the world perhaps the most free from septic hospital contamination. (Med. and Surg. Trans., Vol. XLVII., p. 75, 1864.)

It is probable that amputation at the hip joint, and at the upper part of the thigh, will be found to undergo a great increase of danger at the age of puberty, when the pelvic organs assume their adult development. This rule probably applies to all operations and injuries to the pelvis and the parts immediately adjacent.

Sex.—The opinions and figures on the influence of sex are in utter contradiction to each other, showing the present unfinished state of our knowledge on plain points which ought long ago to have been well settled. The Dictionnaire des Sciences Médicales, in the article on amputations, says that women bear amputations better than men, and cites in proof, the statistics of Newcastle, Glasgow, Edinburg and Paris, summed up as follows:

| | CASES. | DIED. | PER CENT. MORTALITY. |
|---|---|---|---|
| Amputations in males | 1244 | 441 | 35 |
| " in females | 284 | 83 | 29 |

On the opposite side, Mr. Callender (Med. and Surg. Trans.,

1864,) says that the mortality of the operation among women is worse by fifty per cent. or more than among men.

Schmidt's Jahrbücher says that at Jenna the mortality of certain amputations was $21\frac{3}{7}$ per cent. worse among the women than among the men.

There is no reconciling such absolute contradictions. All we can say is that at present the influence of sex on the mortality has not been properly determined.

Time of Operation.—Here we are met again with irreconcilable contradiction. Dr. Ashurst, of Philadelphia, has tabulated in his Surgery, p. 110, 2,201 cases from the civil hospitals of both continents, showing primary amputations of all kinds as having a mortality of 32 per cent., and secondary ones of 50 per cent. The statistics of military amputations in the Crimean and American wars show a similar result.

On the other hand, the Dictionnaire des Sciences Médicales, tome III., p. 770, gives figures which foot up as follows:

| | CASES. | DIED. | PER CENT. MORTALITY. |
|---|---|---|---|
| Primary amputations of all kinds | 5599 | 3164 | 56.51 |
| Secondary " " " | 2265 | 1290 | 56.95 |

These statistics show the primary and secondary cases to have almost exactly the same mortality.

The usual assertion of authors is that, in general, primary amputations are safer than secondary, but that at the hip and in the upper half of the thigh secondary ones are the safest.

The greater danger of primary than of secondary amputations at the hip and upper part of the thigh, is established, at least in adults. It is not proved, nor provable in children, who bear operations about the hips much better than adults; but the vast mass of figures above quoted show that, taking all kinds together, primary amputations have scarcely a shade of superiority over the secondary, a result in bold contradiction to the opinions taught by nearly all authors.

Intermediary Amputations.—Military surgeons divide the cases ordinarily called secondary into two parts, those performed after the first 24 hours, and before the establishment of free suppuration, being separated from the remoter secondary, and termed *intermediary*. These are considerably more fatal than either primary or secondary operations, except in the case of the hip joint. At that articulation every stage is safer than the primary.

The underlying principle appears to be that the presence of acute inflammation is a source of greatly increased danger, and large operations should be avoided if possible during its existence.

Pathological Condition.—Here is a wide field of investigation, which has been only imperfectly explored. Traumatic amputations on the average are much more fatal than those performed for disease. Ashurst's table (Surgery, p. 109,) collates over 4,000 cases of all kinds, and gives;

Mortality of traumatic amputations,....41 per cent.
" " pathological " ....30 "

But all traumatic cases are not alike. Those which have produced a chronic trouble of many months' duration, often become to all intents and purposes like pathological cases.

In suppurating knee joints, both traumatic and pathological, the acute stage of the inflammation is an excessively dangerous time for amputation, and is to be avoided by all possible means.

Amputations of "complaisance," or "expediency," so-called, that is, amputation of members otherwise healthy, but simply deformed, though technically classed as pathological, have not the safety of other pathological cases. Their rates of mortality are almost the same as those of traumatic cases.

Cancer, necrosis, caries of joints, etc., are pathological conditions often demanding amputation, and the danger of one perhaps differs from that of another, but the literature of the profession gives only an obscure light respecting it. At present we know little of the difference, but only the general fact that taken together, amputations for these causes are much safer than those performed for traumatic causes.

Flap or Circular Operations.—Efforts were formerly made to show statistically an advantage of one or the other methods, but the results were contradictory, and with the multiplication of new plans the old discussion between the advocates of flap and circular amputations died out, without any decided superiority being shown for either. At the present day surgeons selecting one or the other method, do it on other grounds than any supposed general difference of mortality between them.

INDICATIONS FOR AMPUTATION.—The Dictionnaire des Sciences Médicales (Article Amputation,) sums up the indications with excellent judgment, and substantially as follows:

*Pathological Causes.*—1, irremovable cancer; 2, diseases of bones not otherwise removable; 3, caries of joints after white swelling (this does not always require it); 4, diffuse aneurism, threatening gangrene, not amenable to ligation; 5, other aneurisms disorganizing parts too seriously to admit of cure by ligature, etc.

*Traumatic Causes.*—6, tearing off of limbs; 7, crushing of both bones and soft parts to disorganization; 8, comminuted fractures, with destruction of the great nerves at the root of the limb; 9, wounds of large joints not admitting of resection and extensive injury of coverings; 10, compound dislocations with great destruction of soft parts and principal vessels; 11, very destructive burns: 12, traumatic gangrene.

Surgeons should carefully avoid the vulgar error of being influenced by the external appearance of the wound, instead of the condition of the parts, and the state of the innervation and circulation.

### RESECTIONS.

Of these important operations I find a considerable number of records, several of which are derived from a valuable report of Dr. Henry Lyster to the Michigan State Medical Society:

### TABLE XII.

### EXSCISIONS OF LARGE JOINTS AND BONES IN THE LAKE STATES.

| NO. | OPERATOR. | Age. | REASON FOR OPERATION. | COMPLICATIONS. | OPERATION. | Cond. at opr | Time to Operation. | Result. | Time to death or recovery. | Practice. |
|---|---|---|---|---|---|---|---|---|---|---|
| 7054 | Dr. E. Andrews | 14 | Caries of ankle | None | Excision of ankle | Good | | recovered | | Hospital. |
| 7510 | " " | 55 | Deformity of ankle fr.inj'ry | " | " " | " | several yrs. | " | | Private |
| 7512 | " " | 40 | Comp. fracture of elbow | " | " elbow | " | primary | " | | " |
| | " " | 7 | Caries of, ankle | " | " ankle | " | 2 years | " | | " |
| | " " | 45 | Caries of knee (recent) | " | " knee | Bad | 2 weeks | died | 10 days | " |
| 6461 | " " | 25 | " shoulder | " | " shoulder | Med | some mos. | recovered | | Hospital. |
| 518 | " " | 23 | " knee | " | " knee | " | " | " | | " |
| | " " | -- | " shoulder | " | Exc. up. 3½in. humerus | " | 2 years | " | | " |
| 5541 | " " | -- | " knee | " | " of knee | " | " | failed | | " |
| 6682 | " " | 19 | " tarsal bones | " | " of ant. tarsals | Good | some mos. | recovered | some ms. | " |
| 483 | " " | 27 | Talipes varus | " | " of ankle | " | congenital. | " | " | " |
| 1977 | " " | 22 | Hip dis. and caries of ankle | Abscess at ankle | " head of femur | Bad | 6 years | died | 4 weeks | " |
| | " " | 20 | Caries of ankle after fract. | None | " of ankle | " | secondary | recovered | 8 mos | Private |
| | " " | 7 | " " " | " | " " | " | some mos | " | | Hospital. |
| 374 | " " | 11 | Hip disease and caries | " | " head of femur | " | 7 years | " | 2 years | Private |
| 648 | " " | 13 | " " " | Tuberculous family | " hd.&trochanter fem | " | 2 years | died | 10 mos | " |
| 662 | " " | 6 | " " " | None | " head and part trochanter of femur | Good | 2 years | recovered | 8 mos | " |
| 649 | " " | 6 | Hip disease, carious | None | Exc. head of femur | " | 2 years | " | 4 mos | " |
| 661 | " " | 13 | " " " | Cachexy following diphtheria | " " " and part of trochanter. | Bad | 1 year | died | 4 weeks | " |
| 536 | " " | 6 | Hip disease, carious | None | Exc. head of femur | Med | 6 mos | recovered | 7 mos | Hospital. |
| 5688 | " " | 25 | Hip disease, carious | None | " " " and 2 in. of trochanter | Bad | several mos | died | 5 days | " |
| | Dr. J. S. Sherman. | 18 | " " " | " | Exc. head of femur | " | 3 years | " | 4 mos | Private |
| | " Bogue, | 26 | " " " | | Exc.hd.fem.& trochant. | | 9 mos | " | 4 mos | Hospital. |
| | " D. Brainard | 8 | " " " | | Exc. head of femur | | | recovered | | Private |

TABLE XII.—Continued.

| | | | | | | | | | | |
|---|---|---|---|---|---|---|---|---|---|---|
| 1532 | Dr. E. Andrews | .. | Caries of knee | None | Exc. of knee | Med | 1 year | died | 5 weeks | Private |
| 7543 | " " " | .. | car. head humerus, gunshot | " | " head of humerus | " | 18 mos | recovered | | " |
| | " D. Brainard | .. | Diseased knee | | " knee | | | " | | |
| | " " " | .. | " " | | " " | | | " | | |
| | " " " | .. | " " | | " " | | | " | | |
| | " " " | .. | " " | | " " | | | died | | |
| | " " " | 60 | Tumor of upper jaw | | " entire jaw | | | recovered | | |
| | " " " | 20 | " " " | | " " " | | | " | | |
| | " A. Fisher | 50 | Caries of elbow | Syphilitic | " elbow joint | Good | 4 years | " | | Private |
| | " J. E. Owens | .. | Hip disease | | " hip joint | | | " | | Hospital |
| | Cook Co. Hospital | .. | Disease of lower jaw | | " nearly entire jaw | | | died | | " |
| | Dr. N. Senn | 10 | Hip disease | | " hip joint and 4½ in. of femur | Bad | 16 mos | recovered | 3 mos | Private |
| | Dr. H. Wardner | 26 | Osteo-sarcoma lft sup. max. | | " left sup. maxilla | Good | 18 mos | " | 5 weeks | " |
| | " E. D. Kittoe | 17 | Necrosis of tibia | None | " shaft of tibia | Bad | 3 years | " | | " |
| | " " " | 12 | " lower jaw | " | " half the body of lower jaw | Med | 2 years | " | | " |
| | Dr. E. D. Kittoe | 11 | Necrosis of lower jaw | None | " " " | Bad | 18 mos | " | | " |
| | " M. Gunn | 25 | Caries of head of humerus | " | " head & 3 in hum's | Good | many mos | " | | " |
| | " J. H. Beech | 37 | Caries of hip joint | " | " hd. & troch. femur | Bad | some years | died | | " |
| | " T. A. McGraw | 8 | " " " | " | " " " | Med | | recovered | | Hospital |
| | " H. F. Lystor | 5 | " " " | " | " " " | Good | | " | | |
| | " " " | 7 | " " " | " | " " " | Good | | " | | |
| | " J. F. Miner | 11 | " " " | | " head and part of trochanter of femur | Bad | | " | | |
| | Dr. D. O. Farrand | 9 | Caries of hip joint | | Exc. femur, head and trochanter of femur | | | died | 10 days | |
| 8448 | Dr. E. Andrews | .. | Caries of ankle joint | None | Exc. ankle joint | Med | | recovered | | Hospital |
| 8598 | " " " | 20 | " of shoulder, stabbed | " | " shoulder joint | " | | " | | " |
| 7794 | " " " | 40 | Comp. fract. ankle | Contus. intern. | " ankle joint | Bad | 20 hours | died | | Private |
| 7823 | " " " | 40 | Commin. sup'g fract. ankle | None | " " " | Good | 4 weeks | recovered | | " |

RECAPITULATION.

| | CASES. | DIED. | PER CENT. MORTALITY |
|---|---|---|---|
| Resection of the shoulder joint | 5 | 0 | |
| " " elbow " | 2 | 0 | |
| " " hip " | 19 | 8 | 42 |
| " " knee " | 8 | 3 | 37 |
| " " ankle " | 9 | 1 | 11 |
| " of other parts | 8 | 1 | 12 |
| Totals | 51 | 13 | 25 |

PRIMARY RESECTIONS OF THE SHOULDER JOINT ABROAD.

| AUTHORITIES. | CASES. | DIED. |
|---|---|---|
| Med. and Surg. Hist. War of Reb'n, part II., vol. II. p. 599 | 515 | 160 |
| St. George's Hosp., London | 1 | 1 |
| Circular No. 6, Surg. Gen. U. S. A. | 210 | 50 |
| Gant's Surgery, p. 672 | 59 | 18 |
| Chisholm's Mil. Surg., Confed. Amer. Army | 41 | 13 |
| Deutsch. Zeit. Chir. Bd. 1, 2 und 5 | 15 | 8 |
| Jahresbericht gesammt, Med. Bd. 2, p. 874 | 1 | 1 |
| Warren's Surg., Confed. Amer Army | 3 | 1 |
| Totals | 845 | 252 |

Mortality of primary cases, 30 per cent.

INTERMEDIARY RESECTION OF THE SHOULDER ABROAD.

| AUTHORITIES. | CASES. | DIED. |
|---|---|---|
| Med. Surg., Hist. Rebellion, part II., vol. II., p. 599 | 120 | 104 |

Mortality of intermediary cases, 46 per cent.

INTERMEDIARY AND SECONDARY CASES COMBINED; ABROAD.

| AUTHORITIES. | CASES. | DIED. |
|---|---|---|
| Med. and Surg. Hist. War of Reb'n, part II., vol. II., p. 599 | 316 | 131 |
| Gant's Surgery, p. 672 | 34 | 6 |
| Chisholm's Mil. Surg. Amer. Confed. Army | 29 | 7 |
| Warren's Surgery, " " " | 2 | 1 |
| Arch. klin. Chir., Bd 10 und 13 | 4 | 1 |
| Deutsch. Zeit. Chir., Bd. 1, 2 und 5 | 49 | 13 |
| Billroth's Briefe | 6 | 2 |
| Dr. Herrgolt, Seige of Strassburg | 3 | 1 |
| Totals | 443 | 162 |

Mortality, 37 per cent.

PATHOLOGICAL RESECTION OF THE SHOULDER; ABROAD.

| AUTHORITIES. | CASES. | DIED. |
|---|---|---|
| Gant's Surgery, p. 657 | 80 | 15 |
| Archiv. klin. Chir. Bd. VIII., S. 106, Bd. X., S. 892, 1893 | 9 | 2 |
| Deutsch. Zeit. Chir. Lücke, Bd. II., S. 380 | 6 | 1 |
| Statist. des Hôp. de Paris, | 1 | 0 |
| Totals | 96 | 18 |

Mortality, 19 per cent.

CONDITIONS IMPERFECTLY STATED; ABROAD.

| AUTHORITIES. | CASES. | DIED. |
|---|---|---|
| Otis' collection of foreign military cases, Med. and Surg. Hist. War of Rebellion, part II., vol. II., p. 607 | 378 | 156 |
| London Hosps., | 8 | 2 |
| Trans. Ill. State Med. Society, 1863 | 6 | 1 |
| Bericht k. k. allg. Krankenhaus, Wien | 6 | 4 |
| Archiv. klin. Chir. Bd. VIII., Bd. X., Bd. XI. | 28 | 9 |
| Heyfelder Lehrbuch Resectionen, p. 210, being cases of Jägers, Paulo, Baudens, Esmarch, Ritter, Beith, Blackman and G. Meyer | 169 | 30 |
| Totals | 595 | 202 |

Mortality, 34 per cent.

SUMMARY.

| | CASES. | DIED. | PER CENT. MORTALITY |
|---|---|---|---|
| Total resections of shoulder abroad | 1979 | 634 | 32 |
| " " " in Lake States | 5 | 0 | 0 |

OPINIONS OF AUTHORS.

Gant's Surgery, p. 656, advocates resection of the shoulder in destructive diseases of the articulation.

T. Holmes, Syst. of Surgery, Vol. V., p. 664, says this operation is to be preferred to amputation in gunshot fracture and compound dislocation of the joint, when the injury is not too extensive, and is the only operation admissable in chronic disease of the joint, except perhaps rapidly growing tumor of the head of the bone, when he prefers amputation.

Billroth in his Surgical Pathology, p. 472, says it is safer than amputation.

Dr. Hodges, of Boston, in his excellent monograph says the results are excellent, but that the limb resulting being no better than that after natural anchylosis, the operation is not to be performed when there is good chance of natural recovery.

T. Holmes, of England, repeats the same opinion.

Bryant, of London, and Woodward, of Washington, (Cir. No. 6, S. G. O.) praise the operation, but Bryant's quotations of Cir. No. 6 are very inaccurate as to this point.

Ashurst says it ought not to be done for malignant disease.

The other authors for the most part accept it as an established and valuable operation, without special discussion.

Löffler (General-Bericht, 1867, S. 288,) favors it in military cases, but condemns it in the intermediary period.

Stromeyer, Schwartz, McLeod and Demme all favor the operation in military surgery.

CONCLUSIONS.

The five shoulder resections in the Lake States were all successful. In other regions the operation is fully recognized as far safer than amputation at the same point. The foregoing table of the operation abroad give as follows:

Average mortality of resection of shoulder joint, 32 per cent.
" " amputation at " " 39 "

Every motive, both of safety and of usefulness of the member requires resection to be preferred to amputation, whenever the conditions admit of the choice. Destruction of the head of the humerus and of considerable portions of the soft parts do not necessitate the loss of the limb. The decision rests mainly on the condition of the axillary nerves and vessels. Generally if, after an injury, there is a pulse at the wrist and some innervation in the hand, the resection is to be preferred.

In old irreducable dislocations with the head of the bone pressing on the axillary plexus, resection has been practiced, but in the present state of surgery a subcutaneous division of the ligaments to any extent necessary to allow of reduction, would usually be preferred.

The diseased conditions requiring the operation are for the most part so plain as to require no discussion.

The primary stage has the least mortality, the secondary the next, and the intermediary period is decidedly the worst, and should be avoided whenever it is possible.

The classified results foot up as follows:

Mortality of primary cases..................30 per cent.
" of intermediary cases............46 "
" of interm. and second. cases comb..37 "
" of pure secondary cases..........29 "
" of pathological cases.............19 "

RESECTION OF THE ELBOW JOINT.

Of this operation my Lake State tables furnish me only two cases, both of which were successful. Abroad the figures were as follows:

PRIMARY RESECTION OF THE ELBOW ABROAD.

| AUTHORITIES. | CASES. | DIED. |
|---|---|---|
| Med. and Surg. Hist. War of Rebel'n, pt. II., vol. II., p. 845 | 318 | 68 |
| Rept. Boston City Hosp., Dr. Cheever | 2 | 2 |
| Warren, Confed. Amer. Army | 1 | 1 |
| Esmarch, quoted in Gant's Surgery, p. 675 | 11 | 1 |
| St. George's Hospital, London | 1 | 1 |
| Chisholm's Mil. Surg., Amer. Confed. Army | 25 | 3 |
| Arch. klin. Chir., Billroth's collection, Bd. X., S. 892 | 4 | 2 |
| " " " Bd. XIII., S. 576 | 1 | 0 |
| Deutsch Zeit. Chir., German-French War, Bd. I., S. 187, und Bd. II., S. 105 | 12 | 3 |
| Siege of Strassburg, 1870–71. Dr. Herrgolt | 4 | 1 |
| Totals | 379 | 82 |

Mortality, 22 per cent.

INTERMEDIARY RESECTIONS OF THE ELBOW ABROAD.

| AUTHORITIES. | CASES. | DIED. |
|---|---|---|
| Med. and Surg. Hist. War of Rebellion, pt. II., vol. II., p. 845 | 196 | 69 |

Mortality, 35 per cent.

INTERMEDIARY AND SECONDARY RESECTIONS OF THE ELBOW ABROAD, COMBINED.

| AUTHORITIES. | CASES. | DIED. |
|---|---|---|
| Med. and Surg. His. War of Rebellion, pt. II., vol. II., p.845 | 250 | 74 |
| Warren's Surgery, Amer. Confed. Army, p. 399 | 3 | 2 |
| Esmarch, quoted in Gant's Surgery, p. 675 | 29 | 5 |
| Prof. Billroth, Arch. klin. Chir., Bd. X., S. 892 | 3 | 2 |
| Billroth's Briefe | 1 | 1 |
| Chisholm's Mil. Surg., Amer. Confed. Army | 36 | 6 |
| Deutsch Zeit. Chir., Bd. I., S. 187 and Bd. II., S. 105 | 64 | 16 |
| Dr. Koch, Arch. klin. Chir. Bd. XIII., S. 575–6 | 4 | 1 |
| Dr. Herrgolt, Seige of Strassburg, 1870–71 | 7 | 5 |
| Total | 397 | 112 |

Mortality, 28 per cent.

PURELY SECONDARY RESECTIONS OF THE ELBOW ABROAD, (LATER THAN INTERMEDIARY.)

| AUTHORITIES. | CASES. | DIED. |
|---|---|---|
| Medical and Surgical History of the War of the Rebellion, part II., vol. II., p. 845 | 54 | 5 |

Mortality, 9 per cent.

PATHOLOGICAL RESECTIONS OF THE ELBOW ABROAD.

| AUTHORITIES. | CASES. | DIED. |
|---|---|---|
| Boston City Hosp. Rep. Dr. Cheever | 3 | 0 |
| Dr. R. Hodges | 119 | 15 |
| Rostock Hospt. Rept., 1868 | 1 | 0 |
| British Army Med. Repts. | 5 | 1 |
| Gant's collection from British Hospitals | 218 | 22 |
| Heyfelder Lehrbuch der Resectionen, S. 246 | 188 | 23 |
| Statist. des Hôp. de Paris | 5 | 2 |
| Archiv. klin. Chir. Bd. VIII. and X. | 16 | 1 |
| Totals | 555 | 64 |

Mortality, 12 per cent.

RESECTIONS OF THE ELBOW, WITH CONDITIONS IMPERFECTLY STATED.

The following cases have been collected by various authors, whose sources of information are partly the same, so that a portion of the cases are duplicated, but as it is impossible to get access to all the original documents quoted, the duplications cannot be eliminated. The totals, therefore, are too large, but as the duplications affect the cases and the deaths to the same extent, they do not materially vitiate the ratio of mortality:

| AUTHORITIES. | CASES. | DIED. |
|---|---|---|
| Deutsch. Zeit. für Chir. Bd. II., III. and IV | 140 | 25 |
| Archiv. klin. Chir. Bd. III., IV., VIII., IX., XIII., XV. and XIX | 428 | 56 |
| Jahresbericht gesammt. Med. 1871, S. 403 | 1217 | 223 |
| Dr. Billroth, Archiv. klin. Chir., Bd. X. | 30 | 4 |
| Zurich Hosp., 1860–67 | 25 | 3 |
| Dutrelepont's table | 333 | 40 |
| Heyfelder's Lehrbuch der Resectionen, S. 246–7 | 286 | 32 |
| Bericht k. k. allg. Krankenhaus Wien | 23 | 11 |
| Stromeyer at battles of Langensalza and Kirchheingen | 25 | 4 |
| Statist. des Hôp. de Paris | 2 | 1 |
| Gant's collection from British Hosps., Surgery, p. 675 | 19 | 1 |
| London *Lancet*, 1862 | 149 | 33 |
| Circular No. 6, Surg. Gen. U. S. A. | 286 | 16 |
| U. S. Marine Hospt. Reports | 2 | 0 |
| Trans. Ill. State Medical Society, 1863 | 4 | 1 |
| Totals | 2969 | 450 |

Mortality, 15 per cent.

GENERAL SUMMARY OF ELBOW JOINT RESECTIONS ABROAD.

Primary ............................22 per cent. mort.
Intermediary ............................35 " "
Intermediary and secondary combined.....28 " "
Pure secondary (later than intermediary)... 9 " "
Pathological ............................12 " "
Unclassified ............................15 " "

OPINIONS OF AUTHORS.

As usual there has been some conflict among writers over this operation, but in the main they are pretty well agreed in its favor.

Circular No. 6, S. G. O., says that the mortality of it in the United States army was a little greater than that of amputation of the arm, but attributes it to the fact that many of the cases were partial resections which the author, Dr. Woodward, considers more dangerous than complete ones in traumatic cases.

The Medical and Surgical History of the War of the Rebellion (prepared by Dr. Otis,) shows that in the American war the secondary cases were far safer than the primary, but recommends the primary because many cases not operated on are supposed to die before reaching the secondary stage.—Part II., Vol. II., p. 905. Yet on page 829 it gives the total mortality of non-operative cases at only ten per cent., which is less than half that of primary excision. As the non-operative cases are generally military, we cannot infer that operation is to be rejected in bad cases, yet the success of the non-operative treatment suggests a doubt whether many would die while waiting for the advantages of the secondary period. The writer of the work thinks that in our army the substitution of resection of the elbow for amputation above effected no saving of life.

The French armies, according to Chenu's statistics, had a fearful mortality in resections of the elbow, so that Prof. Sédillot declares it ought to be rejected from the service; but the German surgeons in the Schleswig-Holstein war had brilliant success with it.

Hugelshofer, Deutsches Zeitschrift für Chirurgie, Bd. III., S. 8, gives the same opinion, viz.: that partial resections are dangerous, and ought not to be performed. At page 6 of the same article he assumes it as certain that complete resection is safer than amputation above the elbow, and remarks, " be the functional results good or bad, as you will, the preservation of a certain number of human lives, which would have fallen a sacrifice to amputation of the arm, or to conservative treatment, must give the operation the first place in the treatment of wounds of the elbow joint."

Heyfelder, in his Lehrbuch der Resectionen, pp. 246–7, gives statistics showing that partial resections are rather more dangerous than complete ones. In an essay elsewhere published, he says the results are brilliant.

Stromeyer recommends it in gunshot wounds.

Demme and Saltzman give the mortality of conservative treatment for gunshot wounds of the elbow as over sixty per cent., which is nearly three times the danger of resection for similar wounds.

Hanover (Deutsches Zeit. Chir., Bd. III., S. 7,) opposes the operation bitterly, declaring that of sixteen army cases only one succeeded in getting anchylosis, and that when anchylosis was not obtained the limb was useless and burdensome, and the patient prone to desire it amputated.

On the other hand, Hugelshofer says that in most cases a sufficient stiffening of the false joint is obtained to give a useful limb.

Lücke, of Berne, admits that absolutely firm anchylosis is not usually obtained, but says that a loose arm is better than none.

Neudörfer gives the operation a high rank.

Billroth says that of sixteen movable joints in his observation, all were more or less useful.

Bickersteth, of Liverpool, says that of forty cases, thirty-eight survived, and all had very useful limbs,

Gant's Surgery, p. 650, recommends the operation.

Holmes' System of Surgery considers the operation probably more dangerous than amputation of the arm.

Erichsen favors it in proper cases.

Gross' System of Surgery, Vol. II., p. 1085, considers it an established operation.

Bryant, Hamilton and Ashurst all recommend the operation in proper cases.

### CONCLUSIONS.

The opinion of several of the above authors that excision of the elbow is more dangerous than amputation of the arm, is wildly erroneous. The foregoing summaries show that the mortality of the excision varies between nine and thirty-five per cent., while that of amputation of the arm is from twenty to thirty-six per cent. It is evident, therefore, that the consideration of safety is decidedly on the side of excision, if the condition of adjacent parts admits of the choice. In regard to conservative, as compared with operative treatment, there are no figures properly arranged for a decision because the non-operative cases are generally the mildest. The conservative figures are very contradictory. Demme and Saltzman put the conservative treatment in gunshot wounds at sixty per cent., while Billroth observed it in twenty-four cases to be eight per cent. The Medical and Surgical History of the War of the Rebellion, part II., Vol. II., p. 829, has by far the largest mass of conservative figures, viz.: Cases, 924; deaths, 96; mortality, 10 per cent. This seems at first glance a fine result, but when we consider that they were mostly slight wounds, we see that we have no true basis of comparison.

When the bones are pulverized by the passage of a bullet, it would be folly to talk of conservatism.

Slight compound fractures of the joint, and mild cases of caries do best without operation, under Lister's antiseptic injections and dressings, but bad comminution of the bone or extensive necrosis require excision. Amputation only comes in when the destruction of the circulation, or the presence of cancer, or other incurable conditions render the loss of the limb unavoidable.

(To be continued.)

